# Bone remodeling serum markers in children with systemic lupus erythematosus

**DOI:** 10.1186/s12969-022-00717-3

**Published:** 2022-07-27

**Authors:** Sheng Hao, Jing Zhang, Bingxue Huang, Dan Feng, Xiaoling Niu, Wenyan Huang

**Affiliations:** Department of Nephrology, Rheumatology and Immonology, Shanghai Children’s Hospital, School of medicine, Shanghai Jiao Tong University, Shanghai, 200062 China

**Keywords:** Pediatric systemic lupus erythematosus, Receptor activator of nuclear factor-κB ligand, Osteoprotegerin, Vitamin D, Glucocorticoid

## Abstract

**Indroduction:**

SLE is an autoimmune multisystem disease. Glucocorticoid is an irreplaceable medication for SLE. Glucocorticoid and inflammatory mediators impact bone remodeling by OPG/RANKL/RANK signal system, which could lead to osteoporosis. Our aim is to detect the expression of RANKL/OPG in children with SLE, and to preliminarily explore the changes of bone remodeling serum markers in children with SLE.

**Methods:**

Serum RANKL and OPG of 40 children with SLE and healthy children were detected by ELISA, while 25(OH)VitD_3_ was detected routinely. Clinical data of children with SLE were recorded, including gender, age, height, weight, BMI, SLEDAI, duration of the disease, cumulative dose of glucocorticoid, and correlation analysis was conducted with RANKL, OPG and 25(OH)VitD_3_.

**Results:**

Serum RANKL concentrations in SLE group were significantly higher than health group (9.82 ± 7.20 vs. 6.80 ± 4.35 pg/ml and 0.081 ± 0.072 vs. 0.042 ± 0.034, *P* < 0.05) respectively, and the concentrations of OPG and 25(OH)VitD_3_ in serum were significantly lower than health group (156.34 ± 57.33 vs. 189.16 ± 68.70 pg/ml and 43.66 ± 31.27 vs. 59.04 ± 21.56 mmol/L, *P* < 0.05). Serum RANKL in children with SLE was positively correlated with the duration of SLE, cumulative dose of GC(*r* = 0.593, 0.727, *P* < 0.05). And it was negatively correlated with serum OPG and 25(OH)VitD_3_ (*r* = -0.601, -0.469, *P* < 0.05). In addition, serum OPG and 25(OH)VitD_3_ concentrations were inversely correlated with cumulative dose of GC (*r* = -0.66, -0.508, *P* < 0.05).

**Conclusion:**

Low levels of vitamin D_3_ and bone metabolic abnormalities still persist in children with SLE even if the disease is in remission, while serum RANKL level was elevated, OPG expression was reduced. In the case of disease remission, GC is involved in the occurrence and development of abnormal bone remodeling through RANKL/OPG.

## Indroduction

Systemic lupus erythematosus (SLE) is an autoimmune multisystem disease with high disability rate and mortality. Osteoporosis is one of the most common complications of SLE [1]. Bone remodeling is a dynamic equilibrium biological process of the interaction between osteoblast and osteoclast. When the balance of bone remodeling is disturbed, and bone resorption exceeds bone formation, it results in conditions like bone loss and osteoporosis [2].

Many childhood chronic diseases have impact on bone health, such as type 1 diabetes mellitus [3], chronic kidney disease [4], rheumatic arthritis [2], and SLE [1], involving bone remodeling degeneration. GC can contribute to bone deterioration through the suppression of bone formation and osteoclast activity. Paradoxically, GC can also contribute to reduce bone loss by the anti-inflammatory effect that may counteract the negative effect on bone [5]. Thus, more markers of bone remodeling are needed.

Receptor activator of nuclear factor-κB ligand (RANKL) and Osteoprotegerin (OPG) are a pair of important bioactive proteins in regulating the balance of bone metabolism. RANKL binds to RANK on cells of the myeloid lineage and induces diferentiation and activation of osteoclast precursors, which leads to increased bone resorption, while the apoptosis of osteoclasts was inhibited. OPG is a soluble decoy receptor for RANKL which prevents the RANKL-RANK binding and inhibits osteoclast maturation, suggesting that the ratio of RANKL/OPG plays a key role in the process of bone remodeling. A variety of cytokines, hormones, growth factors can regulate the expression of RANKL [6].

Glucocorticoid (GC) and inflammatory mediators could impact bone remodeling by OPG/RANKL/RANK signal system [2]. There have been few studies on the interactions of the OPG/RANK/RANKL system in children with lupus. In this study, we detected RANKL, OPG and 25(OH)VitD_3_ in 40 children with SLE, while correlation between RANKL and other indexes were analysed, to explore the changes of bone remodeling in condition of lupus.

## Participants and methods

### Patients and controls

Forty children with SLE were enrolled, who were treated and followed up in Shanghai Children's Hospital from January 2019 to July 2020, including 5 males and 35 females. All patients were diagnosed according to 2012 Systemic Lupus International Collaborating (SLICC) classification criteria. Systemic lupus erythematosus disease activity indexes (SLEDAIs) of the patients were below 4, which means the disease were in remission. The control group included 40 healthy children who underwent health check-up at the same period, while the age and sex matched. Patients with early onset of lupus-like syndrome due to genetic mutation, SLEDAIs are greater than 4, and severe infection were excluded. The study was approved by the Ethics Committee of Shanghai Children’s Hospital(No. 2020R015), and the informed consent was signed by the patient’s parents. This study was performed in accordance with the Helsinki Declaration of 1964 and its later amendments.

### Experimental detection and other indexes

The whole blood samples of 40 children with SLE and healthy control group were collected and agglutinated for 30 min, then centrifuged at 1000 RPM for 10 min. Serum samples were stored at -80℃. RANKL and OPG were detected by Human Trance/TNFSF11/RANKL ELISA Kit and Human Osteoprotegerin/OPG ELISA Kit(Multisciences Biotech, Hangzhou, China). Serum 25(OH)VitD_3_ was detected by Roche cobas E601 Analyzer. Cumulative dose of GC was the ratio of total dose to body weight at the time of sampling.

### Statistical analysis

SPSS 25.0 statistical software package was used for statistics. Serum RANKL, OPG, 25(OH) VitD_3_ concentrations, age, duration of SLE, height, weight and cumulative dose of GC were expressed as mean ± standard deviation (SD). The comparisons between two groups were used by t-tests, and Pearson analysis was used to analyze the correlation between two indexes in children with SLE.

## Results

### General clinical data

In this study, there were 40 children in SLE group and 40 healthy children in control group, and there was no statistical difference in gender composition and age between the two groups (Table 1).

**Table 1:** General clinical data of children with SLE group and control group (n or mean ± SD)

	SLE group (*n* = 40)	Control group (*n* = 40)	statistics	*P* value
Sex(male/female)	5/35	6/34	***χ2*** = 0.3922	0.5312
Age(years)	10.75 ± 2.56	11.4 ± 1.53	***t*** = 1.378	0.1721

*SLE* Systemic lupus erythematosus

### Serum RANKL, OPG and 25(OH) VitD3 concentrations in SLE group

**Fig. 1:**
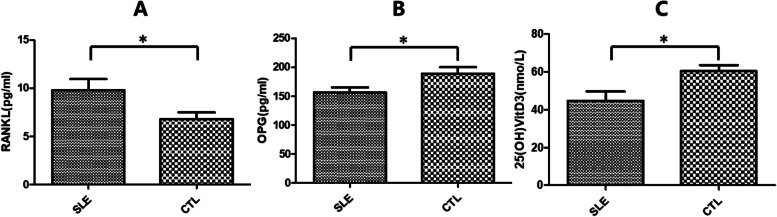
Serum RANKL, OPG, and 25(OH) VitD_3_ concentrations in children with SLE and healthy children(control group, CTL). **A** Serum RANKL concentration in SLE group was significantly higher than control group (*P < 0.05); **B** Serum OPG concentration was significantly lower in the SLE group than control group (*P < 0.05); **C** RANKL/OPG ratio in SLE group was significantly higher than control group (*P < 0.05)

Serum RANKL concentrations and RANKL/OPG Ratios in SLE group were significantly higher than that in control group respectively (*P < 0.05), while the serum concentrations of OPG and 25(OH) VitD3 were significantly lower than those in control group (*P < 0.05), the difference was statistically significant (Table 2, Fig. 1).

**Table 2:** Serum concentrations of RANKL, OPG and 25(OH) vitD_3_ in SLE and control group (n or mean ± SD)

	SLE group (*n* = 40)	Control group (*n* = 40)	Statistics	*P* value
RANKL(pg/ml)	9.82 ± 7.20	6.80 ± 4.35	t = 2.276	0.0256^*^
OPG(pg/ml)	156.34 ± 57.33	189.16 ± 68.70	t = 2.319	0.0230^*^
25(OH)vitD_3_(mmol/L)	43.66 ± 31.27	59.04 ± 21.56	t = 2.712	0.0082^*^

*RANKL* Receptor activator of nuclear factor-κB ligand, *OPG* Osteoprotegerin

^*^P < 0.05

### Correlation analysis of serum RANKL level and other indexes in SLE group

Serum RANKL concentrations in children with SLE were correlated with other indexes, including age, duration of disease, weight, height, Body mass index (BMI), cumulative dose of GC, OPG and 25(OH) VitD_3_. The results showed that in children with SLE serum RANKL was positively correlated with the duration of SLE and the cumulative dose of GC (*P < 0.05), and was negatively correlated with serum OPG and 25(OH) VitD_3_ (*P < 0.05), while not correlated with height, weight, BMI and age (*P* > 0.05). In addition, serum 25(OH) VitD_3_ concentrations were inversely correlated with cumulative doses of GC (*P < 0.05), the difference was statistically significant (Table 3).

**Table 3:** Correlation analysis of serum RANKL level and other indexes in children with SLE(n or mean ± SD

	SLE group(*n* = 40)	*R* value	*P* value
Age(years)	10.75 ± 2.56	-0.105	0.519
Duration of SLE(Months)	14.95 ± 4.94	0.593	< 0.0001^*^
Weight(kg)	42.33 ± 9.68	-0.279	0.081
Height(cm)	131.33 ± 13.92	-0.141	0.385
BMI(kg/m^2^)	24.64 ± 4.88	-0.176	0.278
Accumulated dose of GC (mg/kg)	292.33 ± 67.93	0.727	< 0.0001^*^
RANKL(pg/ml)	9.82 ± 7.20	-	-
OPG(pg/ml)	156.34 ± 57.33	-0.601/-0.66^b^	< 0.0001^*^
25(OH)vitD_3_(mmol/L)	43.66 ± 31.27	-0.469/-0.508^b^	0.002^*^/-0.001^*^

*SLE* Systemic lupus erythematosus, *RANKL* Receptor activator of nuclear factor-κB ligand, *OPG* Osteoprotegerin, *BMI* Body Mass Index, *GC* Glucocorticoid

^*^P < 0.05, the difference was statistically significant

^b^ The correlation with accumulated dose of GC

## Discussion

The vast majority of children with SLE need lifelong treatment, and the mortality is still high in young patients [7]. SLE is prone to recurrence and injury kidney, cardiovascular, nervous system and other organs, so it harms children's physical and mental health, and places a heavy burden on families and society. GC is widely used in patients with inflammatory, autoimmune and allergic diseases, which is one of the most common and irreplaceable drugs for SLE at present. More than 80% of patients with SLE need long-term maintenance treatment [8, 9]. In recent decades, the mortality of SLE patients has decreased significantly and the survival time has been greatly prolonged, mainly due to the use of GC and other immunosuppressants. The chronic complications like bone problem caused by medications and disease itself get more attention. Osteoporosis and fragility fractures occur frequently among SLE patients, including juvenile patients [1].

The ratio of RANKL/OPG plays a key role in the process of bone remodeling. Therefore, RANKL and OPG may become new sensitive biomarkers to evaluate bone metabolism in children. Many studies have shown that RANKL and OPG were changed in SLE and other immune-related diseases (Table 4). Ali et al. found that OPG, RANKL and RANKL/OPG were significantly increased in the SLE group, and OPG level was related to the activity of the disease [10]. But another study on children with SLE showed that RANKL level was not related to disease activity [11]. Therefore, our study selected the patients with disease activity controlled to reduce the interference of inflammatory activity with RANKL/OPG. The results showed that the significantly dfferences in serum RANKL and OPG concentrations between children with disease remission and normal (healthy) children. It suggested that dynamic balance regulation of bone metabolism still persisted even in the remission state, partially proved that RANKL is not related to disease activity. But the effect on bone in the active phase of the disease still needs to be further explored and clarified.

**Table 4:** Previous literature on changes of RANKL and OPG in SLE and other immune-related diseases

Authors	Publication year	Region	Disease	Patients /Controls NO	Age years	RANKL/OPG
Ali et al. [10]	2019	Egypt	pSLE	50/50	11.8 ± 2.99	RANKL/OPG ratio was elevated in pSLE
Sandal et al. [11]	2017	India	pSLE	31/0	13.4 ± 3.2	No different between active and inative disease
Gupta et al. [12]	2016	India	LN	127/24	12–50	Urinary OPG/Cr was elevated in patients with LN
Lien et al. [13]	2010	Norway	JIA	90/90	10.1 ± 3.2	RANKL/OPG ratio was elevated in JIA
Wasilewska et al. [14]	2010	Poland	INS	90/70	10.6 ± 5.5	RANKL/OPG ratio was elevated in INS
Ozkaya et al. [15]	2007	Turkey	CKD	33/22	5–18	OPG was elevated and RANKL was decreased in CKD
Rouster-Stevens et al. [16]	2007	US	JDM	37/44	6.3 ± 2.4	RANKL/OPG ratio was elevated in JDM

*pSLE* Pediatric systemic lupus erythematosus, *RANKL* Receptor activator of nuclear factor-κB ligand, *OPG* Osteoprotegerin, *LN* Lupus nephritis, *Cr* Creatinine, *JIA* Juvenile idiopathic arthritis, *INS* Idiopathic nephrotic syndrome, *CKD* Chronic kidney disease, *JDM* Juvenile dermatomyositis

GC stimulates RANKL expression and inhibit OPG expression, which promotes osteoclast differentiation and osteolysis, while it induces osteocyte apoptosis and inhibits osteocyte generation [17]. Bone loss caused by the GC can be roughly divided into two stages, bone mineral density loss fast which is about 6 ~ 12% in the first year of treatment, and about 3% a year later [18]. At the later stages, GC increased apoptosis of osteoblasts and osteocytes, and then the expression of RANKL decreased [19], while the number of osteoclasts decreased by apoptosis and autophagy. Then bone loss was attenuate at the later stage as the disease got controlled and the GC dosage was reduced. Gupta et al. found that increased urine OPG level in active Lupus Nephritis (LN) and consequently decreased when the disease was controlled, suggesting that OPG may be increased by inflammatory stimulation, but decreased by GC [12]. Our study showed that RANKL expression was increased while OPG expression was decreased in the remission state in children with SLE, and respectively they were positively and negatively correlated with cumulative dose of GC. It proved that bone remodeling markers could be affected by GC dosage. Since the durations of the disease in our patients are all less than 2 years, longer follow-up and observation are needed. It is hard to distinguish whether the observed differences were present due to disease itself or glucocorticoids, despite exclusion of disease activity at the time of sampling. Thus, we should collect more samples of patients with SLE and other autoimmune diseases when they are not treated with glucocorticoids and dynamically detect the changes of the markers.

The correlation between BMD and RANKL/OPG levels is still inconclusive, but the correlation of increased RANKL/OPG ratio and BMD has been confirmed in many childhood diseases [13–15, 20]. SLE may lead to decreased BMD, but the effect of RANKL and OPG on BMD in active stage of SLE is not clear. In our study, all the 40 children with SLE were in remission, so chronic inflammation in SLE might have little effect on RANKL and OPG. Meanwhile, the correlation between GC accumulation and RANKL/OPG suggested that GC played a very important role in regulating the balance of RANKL and OPG, while other blood biochemical indexes were not correlated with them. At present, the most commonly used method for BMD assessment is dual-energy (DXA). But local DXA is insufficient to reflect the condition, and the whole body DXA is difficult to perform in children because of the high dose of radiation and cooperation problem in young children. Considering that, BMD was not performed in these cases, which is a limitation and the correlation between RANKL/OPG and BMD cannot be determined. Thus, further animal and cell studies are warranted to find out the potential machanisms of the bone loss by GC in SLE model.

Vitamin D is a marker of bone metabolism, which regulates the balance of calcium and phosphorus. Vitamin D also has immunomodulatory functions, which plays an important role in both infectious and autoimmune diseases [21]. Serum 25(OH)VitD_3_ level is considered to be the best reflection of vitamin D status. Low vitamin D level is a risk factor of osteoporosis. Our study also found that children in SLE with have low vitamin D levels, which were correlated with cumulative dose of GC, RANKL and OPG levels. It speculated that GC may participate in bone remodeling process in SLE mediated by the transformation of RANKL/OPG, which characterized by low levels of vitamin D status. But the specific regulatory mechanism still needs further research.

## Conclusion

Low levels of vitamin D_3_ and bone metabolic abnormalities still persist in children with SLE even if the disease is in remission, while serum RANKL level was elevated, OPG expression was reduced. These changes are associated with duration of SLE and cumulative dose of GC. It states that GC is involved in the process of the occurrence and development of bone metabolic abnormalities through RANKL/OPG in condition of disease remission.

## Data Availability

Please contact the corresponding author for data requests.

## Abbreviations

SLE: Systemic lupus erythematosus
GC: Glucocorticoid
RANKL: Receptor activator of nuclear factor-κB ligand
OPG: Osteoprotegerin
RANK: Receptor activator of nuclear factor-κB
SLICC: Systemic Lupus International Collaborating
SLEDAI: Systemic lupus erythematosus disease activity index
LN: Lupus nephritis
DXA: Dual energy x-ray absorptiometry
BMD: Bone mineral density
JIA: Juvenile idiopathic arthritis
INS: Idiopathic nephrotic syndrome
CKD: Chronic kidney disease
JDM: Juvenile dermatomyositis
